# Small RNA Deep Sequencing Reveals Role for *Arabidopsis thaliana* RNA-Dependent RNA Polymerases in Viral siRNA Biogenesis

**DOI:** 10.1371/journal.pone.0004971

**Published:** 2009-03-24

**Authors:** Xiaopeng Qi, Forrest Sheng Bao, Zhixin Xie

**Affiliations:** 1 Department of Biological Sciences, Texas Tech University, Lubbock, Texas, United States of America; 2 Department of Computer Science, Texas Tech University, Lubbock, Texas, United States of America; Texas A&M University, United States of America

## Abstract

RNA silencing functions as an important antiviral defense mechanism in a broad range of eukaryotes. In plants, biogenesis of several classes of endogenous small interfering RNAs (siRNAs) requires RNA-dependent RNA Polymerase (RDR) activities. Members of the RDR family proteins, including RDR1and RDR6, have also been implicated in antiviral defense, although a direct role for RDRs in viral siRNA biogenesis has yet to be demonstrated. Using a crucifer-infecting strain of *Tobacco Mosaic Virus* (TMV-Cg) and *Arabidopsis thaliana* as a model system, we analyzed the viral small RNA profile in wild-type plants as well as *rdr* mutants by applying small RNA deep sequencing technology. Over 100,000 TMV-Cg-specific small RNA reads, mostly of 21- (78.4%) and 22-nucleotide (12.9%) in size and originating predominately (79.9%) from the genomic sense RNA strand, were captured at an early infection stage, yielding the first high-resolution small RNA map for a plant virus. The TMV-Cg genome harbored multiple, highly reproducible small RNA-generating hot spots that corresponded to regions with no apparent local hairpin-forming capacity. Significantly, both the *rdr1* and *rdr6* mutants exhibited globally reduced levels of viral small RNA production as well as reduced strand bias in viral small RNA population, revealing an important role for these host RDRs in viral siRNA biogenesis. In addition, an informatics analysis showed that a large set of host genes could be potentially targeted by TMV-Cg-derived siRNAs for posttranscriptional silencing. Two of such predicted host targets, which encode a cleavage and polyadenylation specificity factor (CPSF30) and an unknown protein similar to translocon-associated protein alpha (TRAP α), respectively, yielded a positive result in cleavage validation by 5′RACE assays. Our data raised the interesting possibility for viral siRNA-mediated virus-host interactions that may contribute to viral pathogenicity and host specificity.

## Introduction

Most eukaryotes possess a remarkably conserved RNA silencing system in which double-stranded RNA (dsRNA) precursors are processed into 21- ∼24-nucleotide (nt) small RNAs that regulate the activity of genes, genetic elements, and invading viruses in a sequence-specific manner [1], [2]. The core RNA silencing machinery involves several evolutionarily conserved protein families, including DICER (DCR) or DICER-LIKE (DCL), ARGONAUTE (AGO), and, in some cases, RNA-dependent RNA Polymerase (RDR) [1], [2]. Proliferation and functional diversification of these factors have led to multiple small RNA biogenesis and regulatory pathways in certain lineages such as flowering plants [2]. This aspect has been well illustrated in *Arabidopsis thaliana*, a plant model whose genome encodes four DCLs, six RDRs and ten AGOs. At least four distinct small RNA-generating pathways have been identified in Arabidopsis; each associated with a subset of the core silencing factors [2], [3]. DCL1 and AGO1 are required for the biogenesis and function of micoRNAs (miRNAs), a class of predominantly 21-nt small RNAs arising from characteristic single-stranded hairpin RNA precursors. RDR2, DCL3, and AGO4 or AGO6 are required for the biogenesis and function of heterochromatin-associated small interfering RNAs (siRNAs), a class of predominantly 24-nt nuclear small RNAs arising from dsRNA precursors [1], [4]. DCL2, and RDR6 and DCL4 function in natural *cis*-antisense transcript-associated siRNA (nat-siRNA) and *trans*-acting siRNA (*ta*-siRNA) pathways, respectively [3], [5].

The role of RNA silencing as an ancient antiviral defense mechanism has been firmly established in plants and some animals [1], [6], mainly based on two lines of compelling evidence. First, virus-specific small RNAs are found in infected host cells [1], suggesting that viral RNAs are targeted by the host silencing machinery. Secondly, many viruses encode proteins that suppress activities of the host silencing machinery, strongly suggesting a viral counter-defense strategy evolved during the long-standing virus-host arms race [1], [7]. Numerous virus-encoded, structurally diverse proteins have been shown to exert silencing suppressor activities by targeting the host RNA silencing pathways at specific steps, ranging from generation to function of small RNAs [7]–[9]. In plants, transgenic expression of virus-encoded silencing suppressors has been shown to recapitulate certain disease symptoms, indicating that interference with the endogenous RNA silencing pathways of the host is an important underlying mechanism for viral pathogenicity [7].

Recent studies have also shed light on the host RNA silencing machinery in plants that functions in antiviral defense. Among the four DCLs in Arabidopsis, DCL2 was the first family member shown to play a role in antiviral defense against *Turnip Crinkle Virus* (TCV). Accumulation of virus-derived small RNAs from TCV, but not those from *Cucumber Mosaic Virus* (CMV) or *Turnip Mosaic Virus* (TuMV), was impaired in the Arabidopsis *dcl2* mutant concomitant with its hyper-susceptibility to TCV, suggesting a role of DCL2 in viral small RNA biogenesis [10]. Analysis of viral small RNA accumulation in a combination of Arabidopsis *dcl* double and triple mutants uncovered important and partially redundant roles for both DCL4 and DCL2 in viral siRNA biogenesis [11], [12]. DCL4, which also functions in the RDR6- and suppressor of gene silencing 3 (SGS3)-dependent production of endogenous *ta*-siRNAs [3], turned out to be the major dicing activity which produces 21-nt viral siRNAs [12]. In the absence of a functional DCL4, either in the loss-of-function *dcl4* mutant or when DCL4 activity is blocked by a silencing suppressor such as the TCV capsid protein (p38), the role of DCL2 in antiviral defense was unmasked with the accumulation of DCL2-dependent 22-nt small RNAs as the major viral siRNA species [11], [12]. In addition to DCL proteins, several AGO family members including AGO1, are also thought to be part of the host silencing machinery involved in antiviral defense, presumably through the formation of RNA-induced silencing complexes (RISCs) that target viral RNAs for destruction [12]–[14]. Several virus-encoded silencing suppressors have been reported to suppress AGO1 function [8], [9].

Despite significant advances in understanding virus-induced RNA silencing, several key aspects concerning the biogenesis and function of virus-derived small RNAs in plants remain obscure. For example, the nature of viral RNA molecules that trigger the host RNA silencing pathway remains unclear. Because dsRNA molecules are known to form as the replicating intermediate of an RNA virus, the dsRNA form of a replicating RNA virus was initially assumed to be the silencing trigger in early models, predicting production of equal amounts of sense and antisense viral siRNAs in infected host cells. However, limited molecular cloning of small RNAs derived from several positive-strand plant RNA viruses has revealed a strong strand bias towards the genomic sense strand, which could argue against the idea of genome-length viral dsRNA as being the sole precursor [15]. An alternative model of highly structured, single-stranded viral RNAs as the silencing trigger was proposed [15]. While the strand bias of virus-derived siRNAs has been observed in several cases, a correlation between specific localized secondary structure and small RNA-generating hot spots in a viral genome has not been definitively established [16], [17]. Moreover, although members of the RDR family proteins, including RDR1 and RDR6, play a role in antiviral defense [1], [18], evidence for the direct involvement of RDR proteins in viral siRNA biogenesis has emerged only very recently [19]. RDR1 was shown to be required for accumulation of siRNAs derived from a silencing suppressor-deficient (CMV-*Δ*2b) strain, but not the wild type strain of CMV, suggesting that RDR1 activity could be masked by the 2b protein, a CMV-encoded silencing suppressor [19]. Furthermore, the potential role of viral siRNAs in regulating host gene expression remains largely unexplored in plants [20].

To address these questions, we took a small RNA deep sequencing approach to capture and analyze the viral small RNA profile from host plant cells at an early infection stage. Using a crucifer-infecting strain of *Tobacco Mosaic Virus* (TMV-Cg) and *Arabidopsis thaliana* as a model virus-host system, virus-derived small RNAs were captured from systemically infected tissues of wild type Arabidopsis (Col-0) as well as *rdr1* and *rdr6* mutant plants at 3 days post-infection (dpi). Here we report the first high-resolution small RNA map for a plant virus, revealing features of TMV-Cg-derived siRNA populations indicative of the mechanism of their biogenesis. Both RDR1 and RDR6 were found to play important roles in biogenesis of TMV-Cg siRNAs, supporting a model for viral siRNA biogenesis in plants that involves multiple DCL and RDR factors. In addition, a large set of host genes was predicted as potential targets for posttranscriptional silencing by TMV-Cg-derived siRNAs, revealing a layer of virus-host interactions that may contribute to viral pathogenicity and host specificity.

## Results

### TMV-Cg-derived small RNAs can be captured at an early infection stage by deep sequencing

**Figure 1:**
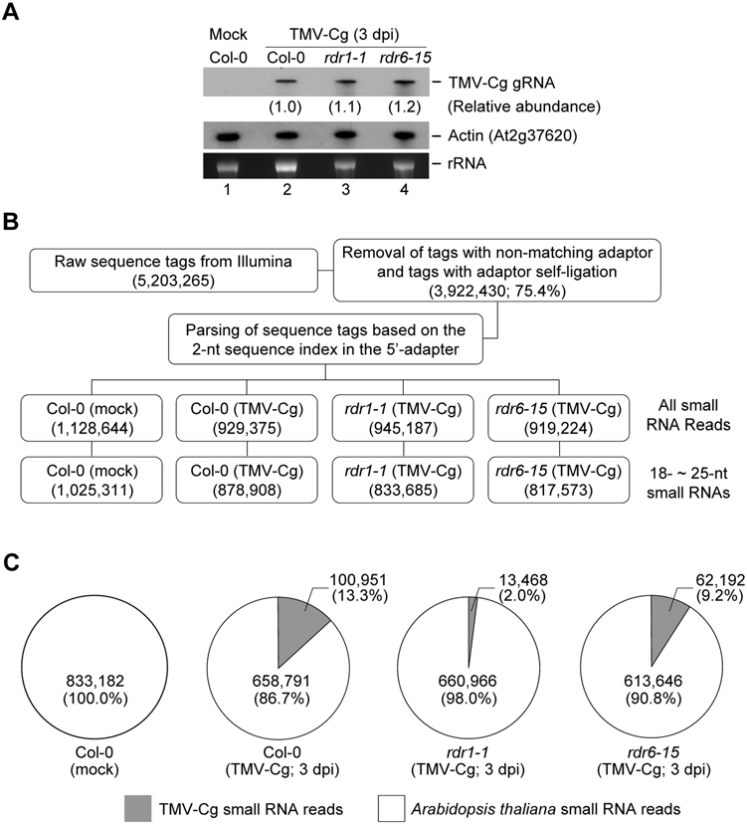
TMV-Cg-derived small RNAs captured from virus-infected wild type Arabidopsis and *rdr* mutants by deep sequencing. (A) Detection of TMV-Cg genomic RNA from infected (3 dpi) leaves by Northern blot assay using a CP-specific probe. The relative abundance (in parentheses) of viral RNA was normalized to signal from the actin probe. (B) A flowchart showing stepwise computational extraction of TMV-Cg-specific small RNA reads from a multiplex small RNA library sequencing sample. (C) Virus- and host-specific small RNA reads recovered from each of the four source libraries.

Profiling of virus-derived small RNAs can help decipher the mechanism for their biogenesis. Recently developed next-generation DNA sequencing technologies offer a cost-effective approach for small RNA profiling [21], [22]. A small RNA deep sequencing approach was adopted that allows analysis of multiplexed small RNA libraries in parallel, to characterize the TMV-Cg-derived small RNA populations in wild type Arabidopsis and *rdr* mutant plants. TMV-Cg infects Arabidopsis systemically and causes mild disease symptoms [23], [24]. TMV-Cg was selected because it induces the expression of *RDR1* in Arabidopsis [24] and provides an opportunity to assess the role of RDR1 and RDR6 in viral small RNA biogenesis. Systemically infected (3 dpi) tissues were used as the source materials for small RNA library construction. By Northern blot using a capsid protein (CP) gene-specific probe, TMV-Cg full-length genomic RNA (gRNA) was readily detectable at 3 dpi in the local inoculated leaves of wild type Arabidopsis (Fig. 1A, lane 2). Slightly elevated levels of TMV-Cg gRNA were detected in *rdr1* and *rdr6* mutants, respectively (Fig. 1A, lanes 3 and 4). These data confirmed successful and uniform infection of TMV-Cg in wild type Arabidopsis and *rdr* mutant plants.

From a pool of four small RNA source libraries constructed in parallel, including virus-infected wild type Arabidopsis (Col-0) as well as the *rdr1* and *rdr6* mutant plants, and mock-infected wild type plants, a total of 5,203,265 raw sequence tags were generated from a single run of Illumina's sequencing-by-synthesis (SBS) platform (Fig. 1B). Removal of sequence tags with a non-matching 5′- or 3′-adapter or resulting from adapter self-ligation yielded a set of 3,922,430 (75.4%) high quality sequence tags, each containing a small RNA insert and perfectly-matched adapters in the expected configuration. The unique indexed sequences engineered with library-specific 5′-adapters allowed unambiguous parsing of the sequence tags among the four source libraries, generating over 900,000 total small RNA reads from each library (Fig. 1B). It is worth noting that the total number of small RNA reads from each of the four source libraries were very similar, ranging from 23.4% (*rdr6* infected with TMV-Cg) to 28.8% (Col-0 mock infected) of the total reads, indicating that an indexed sequencing strategy did not create significant bias among the source libraries (Fig. 1B). Remarkably, the difference in total small RNA reads between any two of the three “TMV-infected” libraries (TMV-Cg-infected Col-0, *rdr1*, or *rdr6*) was less than 2.8%, allowing reliable across-library comparisons to be made for a specific small RNA component. Small RNAs <18-nt or >25-nt in length (9.4% of the total reads) were discarded from further analysis (Fig. 1B). A computational search was performed for each library to select TMV-Cg-derived small RNAs based on a perfect match to the viral genome in either the sense or antisense orientation. While no TMV-Cg-derived small RNA was found in the control library (Col-0; mock), a search in the TMV-Cg-infected wild type library (Col-0; TMV-Cg 3dpi) identified 100,951 viral small RNA reads (Fig. 1C) in sufficient abundance (13.3% of total small RNA reads) to provided an extensive coverage over the relatively small 6,303-nt TMV-Cg genome. These results suggest that the deep sequencing approach can be used as a highly sensitive and efficient method for capturing virus-derived small RNAs from host cells at an early infection stage.

### The TMV-Cg genome harbors multiple highly reproducible small RNA-generating hot spots

**Figure 2:**
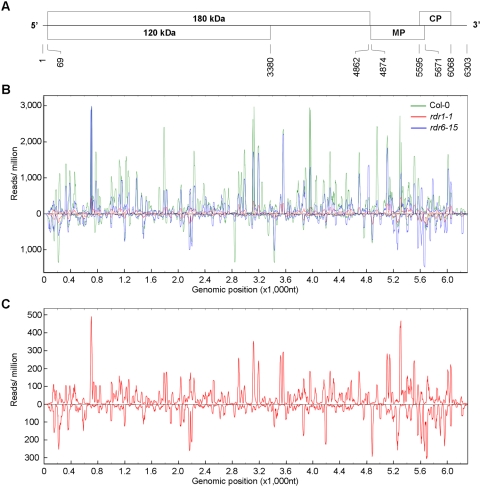
A genome view of TMV-Cg-derived small RNAs captured by deep sequencing. (A) A schematic diagram showing organization of the TMV-Cg genome. The four open reading frames, which encode the ∼120 kDa and the ∼180 kDa replicase proteins, the ∼30 kDa movement protein (MP), and the ∼17.5 kDa CP, respectively, are shown. Numbers below the diagram indicate the genomic coordinates. (B) TMV-Cg-derived small RNAs mapped to the viral genome. Small RNAs were mapped to the viral genome, in either sense (above the X-axis) or antisense (below the X-axis) configuration. The abundance of small RNAs was calculated and plotted as the sum of library-size-normalized reads in each single-nucleotide sliding window along the viral genome. Data from different source libraries are color-coded in *green* (Col-0; TMV-Cg 3dpi), *red* (*rdr1-1*; TMV-Cg 3dpi), and *blue* (*rdr6-15*; TMV-Cg 3dpi). (C) A genomic map of viral small RNAs recovered from the *rdr1* mutant library, viewed with an altered Y axis scale.

To examine the genomic distribution of the viral small RNAs, small RNAs were mapped to the TMV-Cg genome containing four open reading frames encoding the ∼120 kDa and ∼180 kDa replicase proteins, the ∼30 kDa movement protein (MP), and the ∼17.5 kDa CP, respectively (Fig. 2A). A genome view of TMV-Cg-derived small RNAs was generated by plotting the 18- to 25-nt viral small RNAs from the infected wild type library against the viral genome according to their polarity and library size-normalized abundance (Fig. 2B; green traces). Several features were immediately revealed from this genome view. First and foremost, the TMV-Cg-derived small RNAs covered the viral genome in near saturation, with only a few minor gaps including those located at both ends of the genome. Secondly, the majority of viral small RNAs mapped to the viral genome in the sense orientation. Thirdly, the TMV-Cg-derived small RNAs in both the sense and antisense categories displayed a strong non-uniform distribution pattern along the genome, with multiple small RNA-generating hot spots distributed across the entire viral genome (Fig. 2B; green traces). Small RNAs localized to the hot spots were up to several hundred-fold more abundant than many others that were represented by only a single read in the library. For example, among the 6,030 unique 21-nt TMV-Cg small RNAs recovered from the infected wild type library, 1,751 were sequenced only once while 19 others were sequenced more than 300 times. The most abundant 21-nt viral small RNA, TMV-Cg-siR5293 (+) (designated according to its genomic origin and polarity) was sequenced 836 times. Two selected highly abundant 21-nt TMV-Cg-small RNAs including TMV-Cg-siR5239 (+) were detected in Northern blot assays using radio-labeled DNA oligonucleotides as probes (supplementary Fig. S1). Consistent with the deep sequencing results, their antisense counterparts and several other low abundant small RNAs were not detected by Northern blots (supplementary Fig. S1 and data not shown). The huge difference in the abundance among viral small RNAs is therefore unlikely to be a cloning artifact. Interestingly, the small RNA-generating hot spots identified from the infected wild type library largely overlapped with those identified from infected *rdr1* and *rdr6* libraries (Fig. 2B and C, red and blue traces, respectively). The highly reproducible nature of the small RNA-generating hot spots is intriguing, since it suggests that the underlying mechanism for the formation of hot spots most likely involves features that are intrinsic to the viral genomic sequence *per se*. One possibility is that certain regions of the viral RNA may form partially double-stranded secondary structures that are recognized by DCL4 and DCL2 for processing, as was previously proposed for the *Cymbidium ringspot tombusvirus* (CymRSV)[15]. The sequences of multiple 100- to 300-nt TMV-Cg RNA fragments spanning each of the ten selected small RNA-generating hot spots were therefore analyzed for extensive hairpin-forming potential using Mfold [25]. However, we were unable to detect any strong hairpin structure that would account for “hot spot small RNA” in a foldback stem (data not shown). Our results therefore argue against a model with highly structured viral genomic RNA being the precursors of viral siRNAs.

### Both RDR1 and RDR6 play a role in the biogenesis of TMV-Cg-derived small RNAs

To genetically assess the role of RDR1 and RDR6 in viral small RNA biogenesis, we compared TMV-Cg-derived small RNAs captured from the infected wild type library and those infected *rdr1* and *rdr6* mutant libraries, respectively. In contrast to the abundant viral small RNA reads in the infected wild type library, the *rdr1* library (TMV-Cg; 3dpi) contained substantially less TMV-Cg-derived small RNA reads (13,468 or 2.0%), although both libraries contained a similar number of small RNA reads of host origin (Fig. 1C). The *rdr6* library (TMV-Cg; 3dpi) also contained decreased TMV-Cg-derived small RNA reads (62,192 or 9.2%) compared to those in the infected wild type library, although to a much lesser extent compared to the *rdr1* library (Fig. 1C). Since both wild type and the *rdr* mutant plants accumulated similar levels of genome-length viral RNA in the infected leaves (Fig. 1A, lanes 2–4), the decreased number of viral small RNA reads in *rdr1* and *rdr6* mutant libraries is unlikely to be caused by unsuccessful or uneven infection. These results suggest that the Arabidopsis RDR1 and RDR6 play an important role in the formation of TMV-Cg-derived small RNAs.

**Figure 3:**
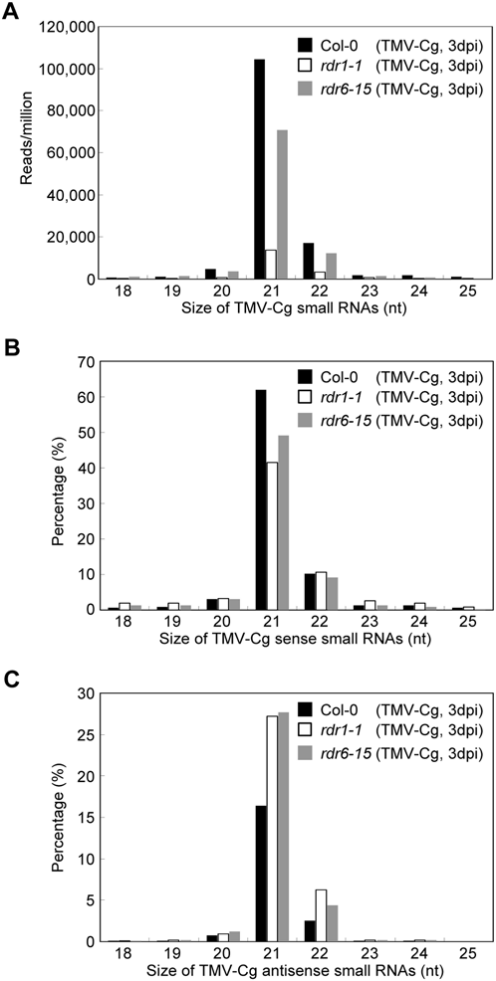
Characterization of TMV-Cg-derived small RNA populations. (A) Size distribution of viral small RNA populations. Proportions of viral siRNAs derived from the genomic sense (B) and antisense (C) strands in each size class are also shown as the percentage of the total viral small RNA reads. Data from different source libraries are represented in black (Col-0; TMV-Cg 3dpi), white (*rdr1-1*; TMV-Cg 3dpi), and grey (*rdr6-15*; TMV-Cg 3dpi) histograms, respectively.

The abundance of TMV-Cg-derived small RNAs recovered by deep sequencing allowed detailed characterization of the viral small populations captured from different genetic backgrounds. We found that the TMV-Cg-derived small RNAs from infected wild type Arabidopsis was predominated by the 21- (78.4%) and 22-nt species (12.9%), with the 21-nt species being, by far, the most abundant (Fig. 3A), indicating that the Arabidopsis DCL4 and DCL2 were the major dicing activities involved in biogenesis of TMV-Cg-derived siRNAs. This observation is consistent with previous reports from small RNA blot-based studies on several other RNA viruses, including TCV, a modified TRV, and CMV [12], [19]. When compared with the infected wild type library, it is obvious that the diminished viral small RNA reads in both *rdr1* and *rdr6* libraries resulted from a partial loss of both the 21- and 22-nt species in the mutant plants, suggesting that both RDR1 and RDR6 function in viral small RNA biogenesis through the DCL4- and DCL2-dependent pathways (Fig. 3A). When the polarity of TMV-Cg-derived small RNAs was examined with respect to the viral genome, nearly 80% of the TMV-Cg-derived small RNA reads in the infected wild type library were found to be “sense”, indicating a strong strand bias of the viral small RNA population. More specifically, the 21- and 22-nt sense viral small RNA reads accounted for 62.0% and 10.3% of the total viral small RNA reads in the library, respectively (Fig. 3B and 3C). The strand-biased feature of the viral small RNA population is consistent with previous observations made in low-throughput sequencing-based studies on CymRSV-infected *Nicotiana benthamiana* and TuMV-infected *Brassica juncea* [15], [16]. Interestingly, the infected *rdr* libraries not only had a reduction in TMV-Cg-derived small RNA reads, but also exhibited a substantially reduced strand bias in the viral small RNA population, with the portion of “antisense” small RNA reads increased to 35.1% and 34.0% in *rdr1* and *rdr6* libraries, respectively. These changes were most prominent in the 21- and 22-nt viral small RNA species (Fig. 3B and C). The ratio of sense/antisense 21-nt viral siRNA reads dropped from 3.77 in the wild type library to 1.52 and 1.78 in *rdr1* and *rdr6* libraries, respectively. Similarly, the ratio of sense/antisense 22-nt viral siRNA reads dropped from 4.02 in the wild type library to 1.71 and 2.06 in *rdr1* and *rdr6* libraries, respectively.

**Figure 4:**
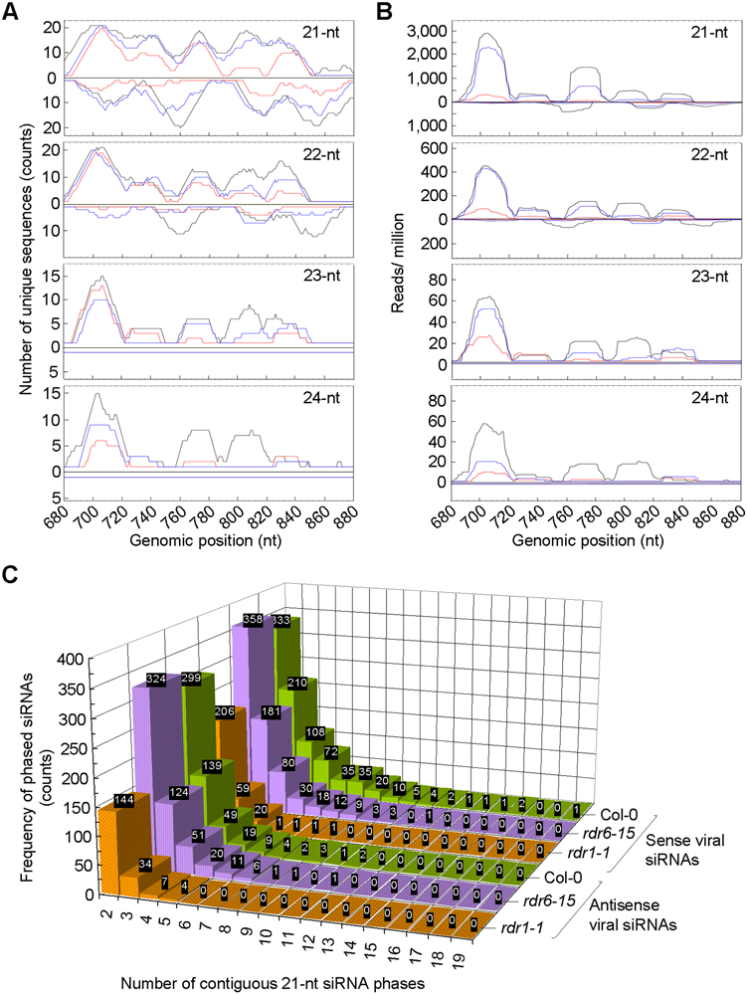
Effects of the *rdr* mutations on TMV-Cg siRNAs biogenesis in *Arabidopsis thaliana*. (A) A close view of a 200-nt TMV-Cg genomic segment showing the number of mapped unique viral siRNAs. A small RNA that is non-identical in sequence to any others within each size class was defined as “unique”. The number of unique siRNAs mapped to a specific genomic location, in either sense (above the X-axis) or antisense (below the X-axis) configuration was plotted in each single-nucleotide sliding window along the viral genome. Data from different source libraries are color-coded in *black* (Col-0; TMV-Cg 3dpi), *red* (*rdr1-1*; TMV-Cg 3dpi), and *blue* (*rdr6-15*; TMV-Cg 3dpi). (B) A close view of the same genomic segment as in (A), showing the abundance of mapped viral siRNAs, which was calculated and plotted as in Fig. 2B. Data from different source libraries are color-coded as in (A). (C) A survey on phased 21-nt TMV-Cg siRNAs in the wild type and *rdr* mutant libraries. Sense and antisense siRNAs are shown separately. The frequencies for phased siRNAs with a defined number (2 to 19) of continued phases are shown.

To gain further insights on viral small RNA biogenesis, the sequence complexity and normalized abundance of TMV-Cg-derived small RNAs were compared for different size classes. Viral small RNAs in each of the 21- to 24-nt size classes were mapped onto the viral genome separately and the small RNA sequence complexity was measured by counting the number of unique small RNA sequences in each of the single nucleotide sliding windows. As shown in a representative 200-nt viral genomic segment, the highest overall sequence complexity of viral small RNAs in the wild type library was found in the 21-nt size class, followed by the 22-nt size class (Fig. 4A, black traces). Notably, in the 21-nt size class, maximum (i.e. 21) or near-maximum sequence complexity was observed for both sense and antisense viral small RNAs at multiple genomic locations, a strong indication that these siRNAs arose from a dsRNA precursor and that DCL4 processing occurred in most, if not all, possible phases (Fig. 4A, black traces). At least at certain locations, comparable sequence complexity for both sense and antisense 22-nt viral siRNAs was observed, suggesting suboptimal processing of the dsRNA precursors by DCL2. This is consistent with the notion that DCL2 functions as a partially redundant secondary dicing activity in antiviral defense. Nonetheless, the fact that many viral siRNAs were found at a very low level and that only a few accumulated to high abundance, mostly the sense siRNAs (Fig. 4B, black traces), suggests the existence of selection mechanism(s) that is not only sequence-dependent but also strand-specific. The low sequence complexity and low abundance of the 23- and 24-nt viral siRNAs were also indicative of their origin from marginal DCL3 activity (Fig. 4A, black traces). A general reduction in sequence complexity across all size classes of viral siRNAs was observed in both the *rdr1* and *rdr6* mutant libraries, with a much more profound reduction found for the most part in the *rdr1* library (Fig. 4A; red and blue traces). Since a general reduction in the abundance of TMV-Cg-derived small RNAs in each size class was also seen in the *rdr1* and *rdr6* mutants (Fig. 4B), we conclude that the reduced total viral small RNA reads in the *rdr* mutants resulted from reduced sequence complexity as well as abundance of the small RNAs.

RDR6 is known to function in an endogenous, DCL4-dependent small RNA pathway which produces phased 21-nt ta-siRNAs from a cleaved RNA template. Since it is possible that RDR6 may function in viral siRNA biogenesis in a mode that is similar to *ta*-siRNA biogenesis, we searched for phased 21-nt TMV-Cg siRNAs in wild type and *rdr* mutant backgrounds. Sense and antisense viral siRNAs in up to 19 and 11 contiguous 21-nt phases, respectively, were found in the wild type library, with longer contiguous phases occurring at lower frequencies (Fig. 4C). The longest contiguous phases for sense and antisense viral siRNAs were substantially decreased to 8 and 4, respectively in *rdr1*, along with decreased frequency of occurrence for all phased siRNAs in this mutant (Fig. 4C). In the *rdr6* mutant, the longest contiguous phase for sense viral siRNAs decreased to 12 although that of the antisense siRNAs remained at 11 (Fig. 4C). Curiously, the occurrence of all phased sense but not antisense siRNAs displayed substantially decreased frequencies in the *rdr6* mutant (Fig. 4C), suggesting a biased effect of the *rdr6* mutation on production of phased viral sense siRNAs. Overall, these data were consistent with a functional role for RDR1 and RDR6 in promoting viral siRNA biogenesis through distinct mechanisms.

### TMV-Cg-derived siRNAs can potentially target a large set of host genes for posttranscriptional regulation

**Figure 5:**
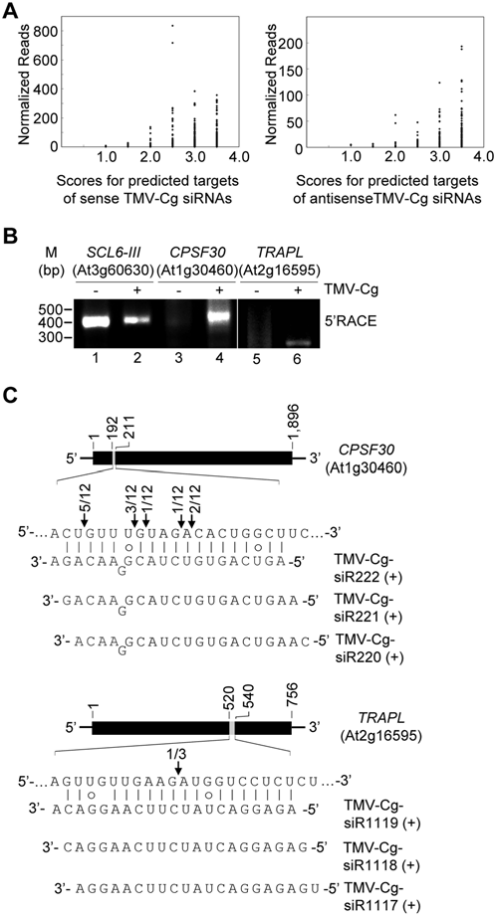
Computational prediction and experimental validation of host genes targeted by TMV-Cg-siRNAs. (A) Scores of host genes predicted as targets for sense (left panel) and antisense (left panel) viral siRNAs. The library size-normalized reads for the associated viral siRNAs are shown. (B) Validation of TMV-Cg-induced cleavage of CPSF30 and TRAP α-like mRNAs by RLM-5′RACE. Gene-specific RACE products for SCL6-III, a known target for miR171, as well as those for CPSF30 and TRAP α-like were resolved on 1.5% agarose gel. M indicates DNA size markers. (C) Sequence alignment between the predicted targets and TMV-Cg siRNAs. Arrows indicate the mapped cleavage sites, and the number of 5′ RACE clones corresponding to each site is shown.

Given the potential sequence complexity of viral siRNAs, it is possible that some of them may target the transcripts of host genes for posttranscriptional regulation. To explore this possibility, an informatics analysis to systemically identify the potential host targets was performed for each of the 21-nt computationally generated TMV-Cg siRNAs. A target prediction algorithm with a scoring stringency similar to those previously used for miRNA target prediction was applied in this procedure (a host gene with a score of 3.5 or lower for a specific viral siRNA was considered as a potential target; see materials and methods) [26]. Based on this procedure, a large set (4,784) of host genes were predicted as potential targets of TMV-Cg siRNAs (Supplementary Table S1). Some host genes were predicted to be targeted by multiple viral siRNAs at distinct locations within the gene. The predicted targets covered a wide range of functional categories, including transcription factors, RNA processing factors, and defense-related proteins (Supplementary Table S1). Intriguingly, we noticed that the TMV-Cg siRNAs with a low-scored predicted host target were in general found at low abundance (Fig. 5A). To test if TMV-Cg siRNAs direct the cleavage of predicted targets *in vivo*, a small subset of the predicted targets were selected for experimental validation by modified RNA ligase-mediated rapid amplification of cDNA ends (RLM-5′RACE) [27], a method that has been widely used for mapping the 5′ end of the 3′ cleavage product. Using the same RNA samples (Col-0; mock and TMV-Cg-infected, 3dpi) as those used for small RNA sequencing, 5′ RACE products were detected for only two of the 16 predicted targets tested. The two host genes yielding a positive 5′RACE product encode a cleavage and polyadenylation specificity factor (CPSF30, At1g30460) and an unknown protein similar to translocon-associated protein alpha (TRAP α), respectively (Fig. 5B). While the miR171-directed cleavage of SCL6-III (At3g60630) mRNA [27] was detected in both mock- and TMV-Cg-infected wild type Arabidopsis (Fig 5B, lanes 1 and 2), cleavage of the CPSF30 and the TRAP α -like mRNAs appeared to be specific to TMV-Cg-infection (Fig 5B, lanes 3–6). Sequencing of 5′RACE products revealed multiple cleavage sites within the predicted TMV-siRNA-interacting region in CPSF30 mRNA, with only one of them corresponded to the predicted canonical site for a cleavage directed by TMV-Cg-siR221(+), one of three viral siRNAs predicted to interact with the target (Fig 5C, upper panel). Sequencing of the TRAP α-like-specific 5′RACE products mapped a cleavage site corresponded to the predicted canonical site for a cleavage directed by TMV-Cg-siR118 (+), one of three viral siRNAs predicted to interact with this target (Fig 5C, lower panel).

**Figure 6:**
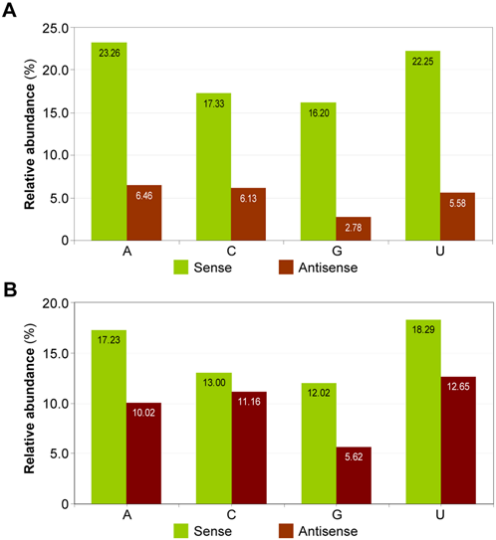
Relative abundance of TMV-Cg-derived 21-nt siRNAs with a distinct 5′-terminal nucleotide. The TMV-Cg-derived 21-nt sense (green) and antisense (red) siRNAs recovered from infected wild type Arabidopsis (Col-0; 3 dpi) were grouped based on their 5′-terminal nucleotide identity. The relative abundance of the viral siRNAs in each group was calculated as the percentage of the total based either on small RNA reads (A) or on the number of distinct small RNA sequences (B).

The lack of a positive 5′RACE result for the remaining 14 predicted targets tested (supplementary Fig. S2) could indicate that not all viral siRNAs produced in vivo are active in directing target cleavage. Since the biological activity of a small RNA depends on the AGO protein to which the small RNA associates, and the 5′-terminal nucleotide identity is known to be an important determinant for small RNAs to form RISC with distinct AGO proteins in Arabidopsis [14], [28], [29], we analyzed the relative abundance of the 21-nt viral siRNAs with a distinct 5′-terminal nucleotide. The TMV-Cg-derived 21-nt siRNAs recovered from infected wild type Arabidopsis were sorted into eight groups according to their polarity and 5′-terminal nucleotide identity. Small RNA reads-based analysis revealed that sense viral siRNAs with a 5-terminal A or U were in general more abundant (23.25% with 5′-A and 22.25% with 5′-U) than those with a 5-terminal C or G (17.33% with 5′-C and 16.20% with 5′-G; Fig. 6A). Antisense viral siRNAs with a 5′ terminal A, C, or U were similarly represented while those with a 5′-terminal G appeared to be underrepresented (Fig. 6A). A “distinct small RNA sequence”-based analysis revealed essentially the same distribution pattern (Fig. 6B). Among the ten Arabidopsis AGO family proteins, AGO1 and AGO4 are known to preferentially recruit small RNAs with a 5-terminal U, whereas AGO2 and AGO5 preferentially recruit small RNAs with a 5′-terminal A and C, respectively [14], [28], [29]. Our data therefore indicate that the TMV-Cg-derived siRNAs likely reside in multiple AGO-containing complexes. Because a “slicer” activity has only been demonstrated for some of the Arabidopsis AGO family members including AGO1, AGO4, and AGO7, it is possible that some viral siRNAs may ends up residing in an AGO family member that does not possess a robust “slicer” activity, which would render the siRNAs incapable of directing target cleavage.

## Discussion

Our work presented here provided the first high-resolution map for small RNAs derived from a plant positive strand RNA virus. Analysis of viral small RNA populations in wild type Arabidopsis and *rdr* mutants led to novel insights on both the biogenesis and function of viral siRNAs in plants.

Several lines of evidence argue against the idea that the bulk of viral small RNAs originate from direct processing of highly structured viral positive strand RNA by DCLs. First, genetic data inferred from dissection of the ta-siRNA pathway suggest that DCL4, which is the major DCL in antiviral defense, produces 21-nt siRNAs from dsRNA substrates instead of other undefined secondary structures [3]. In addition, the only two Arabidopsis miRNAs (miR822 and miR839) that are known to be DCL4-dependent have predicted precursors with an unusually long, near-perfect foldback hairpin structure [30]. Secondly, a correlation between small RNA-generating hot spots and genomic segments with extensive intramolecular dsRNA-forming capacity has not been demonstrated for any viral genome. Our attempt to establish such a correlation for the TMV-Cg genome was also unsuccessful. Finally, results from a recent study in Drosophila, an organism lacking cellular RDR activity, also support a model for viral siRNA biogenesis from dsRNA precursors [31]. Taken together, viral siRNA biogenesis in plants is most likely seeded by DCL4 processing of viral replicating intermediate followed by host RDR-mediated production of secondary siRNAs. Presumably, the level at which a specific viral siRNA accumulates *in vivo* will largely depend on whether it can be efficiently recruited into an AGO complex.

Using a small RNA deep sequencing approach, we demonstrated an important role for Arabidopsis RDR1 and RDR6 in the biogenesis of TMV-Cg-derived siRNAs. The *RDR1* gene and its viroid-inducible expression were first characterized in tomato (*Solanum lycopersicum*) [32]. The partially purified tomato RDR1 was able to catalyze the synthesis of RNA products of over 100 nt in a template-dependent manner *in vitro* [32]. The Arabidopsis and *Nicotiana tobaccum* orthologs of RDR1 are inducible upon TMV infection or salicylic acid (SA) application and play a role in antiviral defense [24], [33]. Interestingly, the *Nicotiana benthamiana* ortholog of RDR1 is an inactive natural variant, which has been postulated to be responsible for *N. benthamiana*'s hyper-susceptibility to many viral pathogens [34]. Consistent with these observations, the Arabidopsis *RDR1* was recently shown to be required for production of viral siRNAs derived from a silencing suppressor-deficient CMV mutant (CMV-Δ2b), suggesting that the role of RDR1 could be masked in plants infected with wild type CMV [19]. How RDR1 functions to promote viral small RNA biogenesis remains unclear. One possibility is that RDR1 may be capable of synthesizing multiple negative-strand RNA fragments from a single positive strand viral RNA template. These short negative strand RNA fragments would then anneal to the positive strand RNAs and promote their processing by DCLs. This speculated role for RDR1 could be significant, especially at an early stage of infection since primary viral siRNAs are likely to be a limiting factor for efficient targeting of viral RNAs for silencing.

*RDR6*, on the other hand, was among the first RNA silencing genes recovered in genetic screens for mutants defective in sense transgene-induced posttranscriptional gene silencing (PTGS) [35], [36]. Curiously, loss-of-function mutations in RDR6 were shown to render Arabidopsis more susceptible to CMV, but not to several other viruses, including a strain of TMV different from the TMV-Cg used in the present study [35], [36]. Works on *N. benthamiana* demonstrated a role for RDR6 in limiting the virus from entering shoot apical meristems, likely through promoting virus-specific secondary siRNA production in systemic tissues [37], [38]. Genetic data from dissection of the *ta*-siRNA pathway in Arabidopsis suggests that RDR6 functions to convert a cleaved transcript into dsRNA, likely in a primer-independent manner [3]. Affinity-purified, epitope-tagged RDR6 was capable of synthesizing long complementary RNA strands using a single-stranded RNA (ssRNA) template *in vitro* [39]. Consistent with the idea that cleaved transcripts may be part of the long-speculated “aberrant RNA” species and recognized by RDR6 to serve as a template, poly (A)-deficient transcripts that arise from a terminator-less transgene were reported to efficiently trigger RDR6-dependent silencing [40]. In light of the recently proposed “two-hit trigger” model for siRNA biogenesis in plants [41], RDR6 likely functions in antiviral defense through formation of viral dsRNA from cleaved, predominantly positive strand viral RNA templates [42]. Subsequent processing of the dsRNAs by DCL4 and DCL2 would then generate a pool of secondary viral siRNAs. In the absence of a functional RDR6, such as in the *rdr6* mutant, an alternative RDR may redundantly function in this postulated pathway, as suggested by the moderate decrease in phased viral siRNA production in the *rdr6* mutant.

Loss-of-function mutations in either RDR1 or RDR6 led to reduced strand bias, as well as reduced overall abundance of the TMV-Cg-derived small RNA population, suggesting that both of these host RDRs play distinct roles in promoting silencing of the TMV-Cg RNAs. An alteration in strand bias of viral small RNAs in *rdr* mutants is rather puzzling, since the assumed function of a host RDR protein is to synthesize dsRNA from ssRNA templates. Subsequent processing of dsRNA by DCLs would yield an equal amount of sense and antisense siRNAs, whose half life in the host cell will largely depend on their selective incorporation into an AGO complex. Perhaps the nascent viral RNA strand resulting from a host RDR activity undergoes certain types of chemical modification such as methylation. While such a modification may not affect processing of the resulting dsRNA by a DCL, it may prevent the modified siRNA strand from being efficiently recruited into an AGO complex. For TMV, the positive strand RNA synthesized during an infection could be a hundred-fold more abundant that the negative strand [43], which would make the positive strand RNA a more likely template for the host RDRs. As a result, the sense viral siRNA would predominate the viral siRISCs. Indeed, viral siRISCs loaded primarily with sense viral siRNAs were observed from plants infected with a tombusvirus [17].

Virus infection can cause a wide range of disease symptoms in a plant host, which is often associated with perturbation of host gene expression [44]–[47]. Some, but not all of the viral disease symptoms could be explained by the virus-encoded silencing suppressor activities which often interfere with the developmentally important host endogenous small RNA pathways [7]. A computational prediction identified a large set of Arabidopsis genes as potential targets for TMV-Cg siRNAs, revealing a novel layer of RNA silencing-based virus-host interactions in plants. This potential regulatory role of virus-derived siRNAs on host gene expression is unlikely to be limited to TMV-Cg. Using the same procedure we have also computationally predicted 2,978 Arabidopsis genes as the potential targets for viral siRNAs derived from TCV (Qi et al., unpublished data), another positive strand RNA virus with a smaller (4,050 nt) genome. We infer from these observations that the potential regulatory role of viral siRNAs on host gene expression is likely common to many cases of virus-host interactions. However, most of the predicted host targets for TMV-Cg-derived siRNAs so far tested failed experimental validation, suggesting that there may be mechanisms that would prevent the viral siRNAs from efficiently targeting a host transcript for cleavage.

We envision that multiple factors could affect the functionality of viral siRNAs and therefore limit their regulatory potential on host targets *in vivo*. While mainly evolved as a viral counter defense strategy, various virus-encoded silencing suppressors could also suppress the activity of viral siRNAs on potential host targets. The replicase subunit of TMV (the 126 KDa protein in common strain; equivalent of the ∼120 KDa protein in TMV-Cg) is a potent RNA silencing suppressor [48] that has been shown to bind miRNA:miRNA* or siRNA duplexes *in vivo* [49], [50] and inhibit RISC formation *in vitro* [49]. Sequestration of TMV-Cg-derived siRNAs by the ∼120 KDa protein could therefore limit the activity of viral siRNAs. In addition, as mentioned earlier, recruitment of viral siRNAs into an AGO protein lacking a robust “slicer” activity could also render the siRNAs inactive in directing target cleavage. This could be the case for viral siRNAs harboring a 5′-termonal G for which a preferred AGO partner has yet to be uncovered. Although a “slicer” activity has not been shown for either AGO2 or AGO5, which is known to preferentially recruit small RNAs with a 5′-terminal A and C, respectively [14], [28], [29], immuno-coprecipitation assay showed that both were able to form complex with CMV-derived siRNAs [14]. Since the TMV-Cg-derived siRNAs that were predicted to target *CPSF30* and *TRAPL*, respectively, harbored a 5′-terminal A or C, we infer that the nucleolytic activities detected by 5′ RACE were likely from AGO2 and/or AGO5. Finally, the extent to which viral siRNAs could modulate host gene expression will also depend on the abundance of functional viral siRNAs. Since the source materials used for viral small RNA analysis in this study were sampled at an early infection stage, a more profound perturbation of host gene expression may be detected at a later stage when viral siRNAs accumulate to a much higher level. It is currently unclear why certain TMV-Cg-derived siRNAs with better-scored predicted host targets were found at low abundance (Fig. 5A). Nonetheless, given the likely widespread potential of virus-host interactions that could be mediated by viral siRNAs, the possibility for such interaction being a contributing factor of viral pathogenicity and host specificity deserves a further look.

## Materials and Methods

### Plant materials and virus infection

The *Arabidopsis thaliana rdr1-1* (SAIL_672F11) and *rdr6-15* (SAIL_617H07) mutant alleles have been described previously [10], [51]. Plants were grown in a commercial soil mix (SunGrow complete mix No. 1; BWI Inc.) in a growth chamber with a cycle of 16 h light at 24°C and 8 h dark at 22°C. Four-week-old plants, typically with 10–11 expanded rosette leaves prior to bolting were used for virus infection. Briefly, five expanded leaves (the 4–8^th^ true leaves) per plant were manually inoculated with purified TMV-Cg (10 µg/mL in 10 mM sodium phosphate buffer, pH 7.5). A parallel set of control plants were mock-inoculated with the buffer following the same procedure. Systemic tissues consisting of leaves and young inflorescences were collected at 3 dpi for RNA extraction. The local inoculated leaves were also collected in parallel for infection analysis. Each sample consists of pooled tissues from a group of 6 plants (from a total of 18 plants).

### RNA preparation and blot assays

Total RNA extraction, purification, and Northern blots were done essentially as described [52]. For detection of full-length TMV-Cg genomic RNA, 2.5 µg of total RNA extracts were gel separated and probed with a ^32^P-labeled 491 bp CP-gene fragment (nt. 5,661–6,151; GenBank accession no. D38444). The blot was stripped and re-probed with a ^32^P-labeled 765 bp cDNA fragment (nt. 402–1,166 from ATG) for Actin.

### Small RNA library construction

Small RNA isolation and library construction were done essentially as described [53], [54]. Modifications were made in the 5′- and 3′- RNA adapter sequences for compatibility with the Illumina's sequencing platform. To allow sequencing from a multiplex library sample, four 5′-RNA adaptors, each with a unique 2-nt sequence “index” (underlined) were used: (1) 5′-AC adapter: 5′- GUUCAGAGUUCUACAGUCCGACGAUCAC-3′; (2) 5′-CA adapter: 5′-GUUCAGAGUUCUACAGUCCGACGAUCCA-3′; (3) 5′-GU adapter: 5′-GUUCAGAGUUCUACAGUCCGACGAUCGU-3′; and (4) 5′-UG adapter: 5′-GUUCAGAGUUCUACAGUCCGACGAUCUG-3′. The sequence of the 3′ RNA adapter which is 5′-monophosphorylated and 3′-idT blocked is: 5′-P-UCGUAUGCCGUCUUCUGCUUGUidT-3′. The final ligation products were PAGE-purified and reverse transcribed (RT) using SuperScript III reverse transcriptase (Invitrogen, Inc), with the DNA oligonucleotides 5′-CAAGCAGAAGACGGCATACGA-3′ as the RT primer. The first-strand cDNA was then PCR amplified for 15 cycles using the Phusion high fidelity DNA polymerase (FINNZYMES, OY; Finland), with the primers 5′-CAAGCAGAAGACGGCATACGA-3′ and 5′-AATGATACGGCGACCACCGACAGGTTCAGAGTTCTACAGTCCGA-3′. The amplified cDNA was PAGE-purified, pooled and submitted to Illumina for sequencing.

### Bioinformatic analysis of small RNA sequences

The small RNA sequences were computationally extracted from the raw sequence tags generated by Illumina and parsed into the four source libraries using Python scripts, based on the 5′-adapter index and the partial 3′-adapter sequence (see Supplementary Materials and Methods S1). For each source library, small RNAs with sequence that perfectly matched the TMV-Cg genome, in either the sense or antisense orientation, were computationally extracted and designated as TMV-Cg-derived small RNAs. The remaining small RNAs with sequence perfectly matching either the Arabidopsis nuclear genome or the organelle genomes were designated as Arabidopsis small RNAs (see supplementary Materials and Methods S1). Small RNAs with a sequence matching neither the virus nor the host genome were discarded without further analysis.

### Computational prediction and experimental validation of host targets for viral siRNAs

A set of 12,566 TMV-Cg-derived 21-nt siRNAs were computationally generated (see supplementary Materials and Methods S1). Putative siRNAs with either low G/C content (G+C<7) or low complexity (a run of 4 or more contiguous “A”s or “U”s) were discarded from further analysis. The TAIR8 cDNA dataset were searched for potential siRNA targets using the TargetFinder software and a mismatch/gap penalty scoring method described previously [26]. Host genes with a score of 3.5 or lower were considered as potential targets. Validation of target cleavage by RLM-5′RACE was done as described previously [27]. The gene-specific primers used in 5′RACE for CPSF30 were CPSF30-645R: 5′-TCTGGCTGACCTGGTGTTGTGATT-3′ and CPSF30-576R 5′-TGGCTGGCCTTGCATTGGAACTT-3′. The gene-specific primers used in 5′RACE for TRAP α-like were TRAPL-780R: 5′-GCCCCCTAACAAAGCGTGTGAGAT-3′ and TRAPL-711R: 5′-ACCCTCAAGCCATTCATCATGCGAA-3′. Primers for SCL6-III were described [27].

## Supporting Information

Supplementary Materials and Methods S1 (0.33 MB PDF)

Table S1 Arabidopsis genes predicted as TMV-Cg-siRNA targets.
(4.98 MB XLS)

Figure S1 Detection of TMV-Cg-siRNAs by Northern blot assays. (A) Northern blot assays with probes specific to TMV-Cg-siR696 (+) (upper panel) and TMV-Cg-siR696 (−) (lower panel). DNA oligonucleotides with sequence corresponding to TMV-Cg-siR696 (+) and TMV-Cg-siR696 (−) were load as positive controls in lanes 1 and 2, respectively. (B) Northern blot assays with probes specific to TMV-Cg-siR5293(+).
(0.12 MB TIF)

Figure S2 Additional predicted TMV-Cg-siRNA targets that were selected for validation by RLM-5′RACE. Sequence alignments between the TMV-Cg siRNAs and the respective predicted targets are shown.
(0.09 MB TIF)

## References

[1] Baulcombe D (2004). RNA silencing in plants.. Nature.

[2] Chapman EJ, Carrington JC (2007). Specialization and evolution of endogenous small RNA pathways.. Nat Rev Genet.

[3] Vaucheret H (2006). Post-transcriptional small RNA pathways in plants: mechanisms and regulations.. Genes Dev.

[4] Vaucheret H (2008). Plant ARGONAUTES.. Trends Plant Sci.

[5] Zheng X, Zhu J, Kapoor A, Zhu JK (2007). Role of Arabidopsis AGO6 in siRNA accumulation, DNA methylation and transcriptional gene silencing.. EMBO J.

[6] Ding SW, Voinnet O (2007). Antiviral immunity directed by small RNAs.. Cell.

[7] Dunoyer P, Voinnet O (2005). The complex interplay between plant viruses and host RNA-silencing pathways.. Curr Opin Plant Biol.

[8] Baumberger N, Tsai CH, Lie M, Havecker E, Baulcombe DC (2007). The Polerovirus silencing suppressor P0 targets ARGONAUTE proteins for degradation.. Curr Biol.

[9] Zhang X, Yuan YR, Pei Y, Lin SS, Tuschl T (2006). Cucumber mosaic virus-encoded 2b suppressor inhibits Arabidopsis Argonaute1 cleavage activity to counter plant defense.. Genes Dev.

[10] Xie Z, Johansen LK, Gustafson AM, Kasschau KD, Lellis AD (2004). Genetic and functional diversification of small RNA pathways in plants.. PLoS Biol.

[11] Bouche N, Lauressergues D, Gasciolli V, Vaucheret H (2006). An antagonistic function for Arabidopsis DCL2 in development and a new function for DCL4 in generating viral siRNAs.. EMBO J.

[12] Deleris A, Gallego-Bartolome J, Bao J, Kasschau KD, Carrington JC (2006). Hierarchical action and inhibition of plant Dicer-like proteins in antiviral defense.. Science.

[13] Morel JB, Godon C, Mourrain P, Beclin C, Boutet S (2002). Fertile hypomorphic ARGONAUTE (ago1) mutants impaired in post-transcriptional gene silencing and virus resistance.. Plant Cell.

[14] Takeda A, Iwasaki S, Watanabe T, Utsumi M, Watanabe Y (2008). The mechanism selecting the guide strand from small RNA duplexes is different among argonaute proteins.. Plant Cell Physiol.

[15] Molnar A, Csorba T, Lakatos L, Varallyay E, Lacomme C (2005). Plant virus-derived small interfering RNAs originate predominantly from highly structured single-stranded viral RNAs.. J Virol.

[16] Ho T, Wang H, Pallett D, Dalmay T (2007). Evidence for targeting common siRNA hotspots and GC preference by plant Dicer-like proteins.. FEBS Lett.

[17] Pantaleo V, Szittya G, Burgyan J (2007). Molecular bases of viral RNA targeting by viral small interfering RNA-programmed RISC.. J Virol.

[18] Wassenegger M, Krczal G (2006). Nomenclature and functions of RNA-directed RNA polymerases.. Trends Plant Sci.

[19] Diaz-Pendon JA, Li F, Li WX, Ding SW (2007). Suppression of antiviral silencing by cucumber mosaic virus 2b protein in Arabidopsis is associated with drastically reduced accumulation of three classes of viral small interfering RNAs.. Plant Cell.

[20] Moissiard G, Voinnet O (2006). RNA silencing of host transcripts by cauliflower mosaic virus requires coordinated action of the four Arabidopsis Dicer-like proteins.. Proc Natl Acad Sci U S A.

[21] Bentley DR (2006). Whole-genome re-sequencing.. Curr Opin Genet Dev.

[22] Lu C, Meyers BC, Green PJ (2007). Construction of small RNA cDNA libraries for deep sequencing.. Methods.

[23] Ishikawa M, Obata F, Kumagai T, Ohno T (1991). Isolation of mutants of *Arabidopsis thaliana* in which accumulation of tobacco mosaic virus coat protein is reduced to low levels.. Mol Gen Genet.

[24] Yu D, Fan B, MacFarlane SA, Chen Z (2003). Analysis of the involvement of an inducible Arabidopsis RNA-dependent RNA polymerase in antiviral defense.. Mol Plant Microbe Interact.

[25] Zuker M (2003). Mfold web server for nucleic acid folding and hybridization prediction.. Nucleic Acids Res.

[26] Fahlgren N, Howell MD, Kasschau KD, Chapman EJ, Sullivan CM (2007). High-Throughput Sequencing of Arabidopsis microRNAs: Evidence for Frequent Birth and Death of MIRNA Genes.. PLoS ONE.

[27] Llave C, Xie Z, Kasschau KD, Carrington JC (2002). Cleavage of Scarecrow-like mRNA targets directed by a class of Arabidopsis miRNA.. Science.

[28] Mi S, Cai T, Hu Y, Chen Y, Hodges E (2008). Sorting of small RNAs into Arabidopsis argonaute complexes is directed by the 5′ terminal nucleotide.. Cell.

[29] Montgomery TA, Howell MD, Cuperus JT, Li D, Hansen JE (2008). Specificity of ARGONAUTE7-miR390 interaction and dual functionality in TAS3 trans-acting siRNA formation.. Cell.

[30] Rajagopalan R, Vaucheret H, Trejo J, Bartel DP (2006). A diverse and evolutionarily fluid set of microRNAs in Arabidopsis thaliana.. Genes Dev.

[31] Aliyari R, Wu Q, Li HW, Wang XH, Li F (2008). Mechanism of induction and suppression of antiviral immunity directed by virus-derived small RNAs in Drosophila.. Cell Host Microbe.

[32] Schiebel W, Pelissier T, Riedel L, Thalmeir S, Schiebel R (1998). Isolation of an RNA-directed RNA polymerase-specific cDNA clone from tomato.. Plant Cell.

[33] Xie Z, Fan B, Chen C, Chen Z (2001). An important role of an inducible RNA-dependent RNA polymerase in plant antiviral defense.. Proc Natl Acad Sci U S A.

[34] Yang SJ, Carter SA, Cole AB, Cheng NH, Nelson RS (2004). A natural variant of a host RNA-dependent RNA polymerase is associated with increased susceptibility to viruses by Nicotiana benthamiana.. Proc Natl Acad Sci U S A.

[35] Dalmay T, Hamilton A, Rudd S, Angell S, Baulcombe DC (2000). An RNA-dependent RNA polymerase gene in Arabidopsis is required for posttranscriptional gene silencing mediated by a transgene but not by a virus.. Cell.

[36] Mourrain P, Beclin C, Elmayan T, Feuerbach F, Godon C (2000). Arabidopsis SGS2 and SGS3 genes are required for posttranscriptional gene silencing and natural virus resistance.. Cell.

[37] Qu F, Ye X, Hou G, Sato S, Clemente TE (2005). RDR6 has a broad-spectrum but temperature-dependent antiviral defense role in Nicotiana benthamiana.. J Virol.

[38] Schwach F, Vaistij FE, Jones L, Baulcombe DC (2005). An RNA-dependent RNA polymerase prevents meristem invasion by potato virus X and is required for the activity but not the production of a systemic silencing signal.. Plant Physiol.

[39] Curaba J, Chen X (2008). Biochemical Activities of Arabidopsis RNA-dependent RNA Polymerase 6.. J Biol Chem.

[40] Luo Z, Chen Z (2007). Improperly terminated, unpolyadenylated mRNA of sense transgenes is targeted by RDR6-mediated RNA silencing in Arabidopsis.. Plant Cell.

[41] Axtell MJ, Jan C, Rajagopalan R, Bartel DP (2006). A two-hit trigger for siRNA biogenesis in plants.. Cell.

[42] Qu F, Ye X, Morris TJ (2008). Arabidopsis DRB4, AGO1, AGO7, and RDR6 participate in a DCL4-initiated antiviral RNA silencing pathway negatively regulated by DCL1.. Proc Natl Acad Sci U S A.

[43] Buck KW (1999). Replication of tobacco mosaic virus RNA.. Philos Trans R Soc Lond B Biol Sci.

[44] Agudelo-Romero P, Carbonell P, de la Iglesia F, Carrera J, Rodrigo G (2008). Changes in the gene expression profile of Arabidopsis thaliana after infection with Tobacco etch virus.. Virol J.

[45] Golem S, Culver JN (2003). Tobacco mosaic virus induced alterations in the gene expression profile of Arabidopsis thaliana.. Mol Plant Microbe Interact.

[46] Havelda Z, Varallyay E, Valoczi A, Burgyan J (2008). Plant virus infection-induced persistent host gene downregulation in systemically infected leaves.. Plant J.

[47] Whitham SA, Quan S, Chang HS, Cooper B, Estes B (2003). Diverse RNA viruses elicit the expression of common sets of genes in susceptible Arabidopsis thaliana plants.. Plant J.

[48] Ding XS, Liu J, Cheng NH, Folimonov A, Hou YM (2004). The Tobacco mosaic virus 126-kDa protein associated with virus replication and movement suppresses RNA silencing.. Mol Plant Microbe Interact.

[49] Csorba T, Bovi A, Dalmay T, Burgyan J (2007). The p122 subunit of Tobacco Mosaic Virus replicase is a potent silencing suppressor and compromises both small interfering RNA- and microRNA-mediated pathways.. J Virol.

[50] Kurihara Y, Inaba N, Kutsuna N, Takeda A, Tagami Y (2007). Binding of tobamovirus replication protein with small RNA duplexes.. J Gen Virol.

[51] Allen E, Xie Z, Gustafson AM, Sung GH, Spatafora JW (2004). Evolution of microRNA genes by inverted duplication of target gene sequences in Arabidopsis thaliana.. Nat Genet.

[52] Xie Z, Allen E, Wilken A, Carrington JC (2005). DICER-LIKE 4 functions in trans-acting small interfering RNA biogenesis and vegetative phase change in Arabidopsis thaliana.. Proc Natl Acad Sci U S A.

[53] Kasschau KD, Fahlgren N, Chapman EJ, Sullivan CM, Cumbie JS (2007). Genome-wide profiling and analysis of Arabidopsis siRNAs.. PLoS Biol.

[54] Llave C, Kasschau KD, Rector MA, Carrington JC (2002). Endogenous and silencing-associated small RNAs in plants.. Plant Cell.

