# Correction: Small RNA Deep Sequencing Reveals Role for *Arabidopsis thaliana* RNA-Dependent RNA Polymerases in Viral siRNA Biogenesis

**DOI:** 10.1371/annotation/8d1a816e-b366-4833-b558-724ec28d1b87

**Published:** 2009-04-01

**Authors:** Xiaopeng Qi, Forrest Sheng Bao, Zhixin Xie

The following information was mistakenly omitted: The complete dataset of TMV-Cg-derived small RNAs described in the manuscript has been submitted to the NCBI's Gene Expression Omnibus (GEO) under the serial number GSE12918.

