# Supplementary material for: Small RNA Deep Sequencing Reveals Role for Arabidopsis thaliana RNA-Dependent RNA Polymerases in Viral siRNA Biogenesis
Source: PLoS One. 2009 Mar 24;4(3):e4971. doi: 10.1371/journal.pone.0004971 (PMC2654919; doi:10.1371/journal.pone.0004971)
Supplement: Supplementary Materials and Methods S1 — (0.33 MB PDF) [file pone.0004971.s001.pdf]

Supporting Information

Supplementary materials and methods

Additional information on bioinformatic analysis of small RNA sequences.

The initial parsing of raw small RNA sequence tags using the Python script "CLASSIFY". To identify the high quality small RNA reads from the raw sequencing data file provided by Illumina, we created a Python script named "CLASSIFY" (see below) to extract the small RNAs sequences with both identifiable 5' and 3' adapter sequences. Sequences tags with a non-matching 5'- or 3'-adapter, with adaptor self-ligation, or with a GFP size marker-specific sequences were removed from further analysis (Fig. 1B). The resulting small RNA tags were then sorted into the four "source libraries" based on the 2-nt sequence index in the 5'-adaptor (Fig. 1B). Small RNAs that are shorter than 18-nt or longer than 25-nt were discarded from each of the four libraries (Fig. 1B).

Sorting of small RNAs of viral and host origin. For each of the four source libraries, small RNAs with a sequence perfectly matching the TMV-Cg genome in either sense or antisense orientation were identified and sorted into a separated bin designated as TMV-Cg-derived small RNAs (Fig. 1C). This was done using the Python script "MAP" (see below). Similarly, small RNAs with a sequence perfectly matching the *Arabidopsis thaliana* nuclear and organellar genomes (version TAIR 8), respectively, were extracted sequentially into distinct bins using the "MAP" algorithm. Of note, from the pool of 18-~25-nt small RNAs, the number of small RNAs matching the nuclear genome were 788,710 (76.9%; Col-0 mock), 628,627 (71.53%, Col-0 TMV-Cg), 616,147(73.9%, *rdr1-1* TMV-Cg), and 575,365 (70.38%, *rdr6-15* TMV-Cg), respectively. Those matching the

organellar genomes were 44,472 (4.3%; Col-0 mock), 30,164 (3.4%, Col-0 TMV-Cg), 44,819 (5.4%, *rdr1*-1 TMV-Cg), and 38,281 (4.7%, *rdr6-15* TMV-Cg), respectively. Small RNAs that match neither the host nor viral genomic sequence were designated as trash and discarded from further analysis. Of note, the number of small RNAs sorted into the trash bin were 192,129 (18.7%; Col-0 mock), 119,166 (13.5%, Col-0 TMV-Cg), 159,251(19.1%, *rdr1*-1 TMV-Cg), and 141,735 (17.3%, *rdr6-15* TMV-Cg), respectively. Computational generation of TMV-Cg-derived of 21-nt siRNAs. The TMV-Cg-derived 21-nt siRNAs were generated *in silico* using the Python script "CUT" (see below). The algorithm generates a total of 12,566 TMV-Cg-derived sense and antisense 21-nt small RNAs from the 6,303-nt genome in all possible phases.

A pseudocode, as well as the actual source code for each of the three Python scripts used in this study is provided below.

Small RNA blot. Small RNA blot assays were done as described previously (Xie et al., 2005). [^{32}P]-end labeled DNA oligonucleotides were used as probes. The probe sequences for each of the TMV-Cg-derived siRNAs were listed below:

5'- ATGAGTTCGGTGCTGCATTGC -3' for TMV-Cg-siR696(-);

5'-GCAATGCAGCACCGAACTCAT-3' for TMV-Cg-siR696(+);

and 5'-TCCAGCTTCAATCCTTAAATT-3' for TMV-Cg-siR5293(+).

Experimental validation of host targets for viral siRNAs. Validation of target cleavage by RLM-5'RACE was done as described previously (Llave et al., 2002). Sequences of the gene-specific primers used in 5'RACE are listed below:

(1) At4g36150_3746R: 5’-CTGTGGGATGATTTGAATAGCCATT-3’ and

At4g36150_3646R: 5’-ACCCGTTGGAAGAATTTTAGCTAACT-3’ for At4g36150;

(2) At1g22870_2866R: 5’-GCACAGAGGCTCTATGATAAATCACA-3’ and

At1g22870_2859R:5’-GCTCTATGATAAATCACAGACGAGAT-3’ for At1g22870;

(3) At2g01390_512R: 5’-CCGGAGCTTGAAACCCAGTGAATCA-3’ and

At2g01390_412R: 5’-TCCGACCGGCTTCTCCGAAGATAT-3’ for At2g01390;

(4) At3g10390_658R: 5’-CGGGTCGTTTCCTACCCTCCAAA-3’ and

At3g10390_603R: 5’-CCTAGCTGCAGCCAACCCAGATA-3’ for At3g10390;

(5) At3g61960_1772R: 5’-CCCGCGGTCTATTTTCGCTAAAGA-3’ and

At3g61960_1743R: 5’-CCGACGATGCTGTAAGTTAGAGAT-3’ for At3g61960;

(6) AT5G51070_2961R: 5’-ACCTCACGGACTAATCACTATGTAT-3’ and

AT5G51070_2784R: 5’-CCGAACCGATGGGTTTCCGGTAT-3’ for At5g51070;

(7) At1g43850_1390R: 5’- GCTCTTCATGCCGCCGAGCACAA-3’ and

At1g43850_1260R:5’- CTCCAGGACAATTTGGCCAGATGAA-3’ for At1g43850;

(8) At5g64550_1947R: 5’- AGCCTTGTTGTTACCACATAGAGA-3’ and

At5g64550_1861R: 5’-GCCCGCCACCATGAACTCTTTCCT-3’ for At5g64550;

(9) At3g54090_654R: 5’- CGCCATCATTTTCCCGTCCTTAAACT-3’ and

At3g54090_578R: 5’- GCTCTCGTCTGAACTCTCTCCTGATT-3’ for At3g54090;

(10) At1g67710_1664R: 5’- GGAAGGTTCTTGGGAAAGCAAGATTA-3’ and

At1g67710_1522R: 5’- CAGGGTCAGGCAGCCGCTCATTT-3’ for At1g67710;

(11) At3g05340_580R: 5’- TCCCCACGGAAATTTCCTTGTCATAA-3’ and

At3g05340_457R: 5’- ACCCACCTGACCCAAGCATTCGTT-3’ for At3g05340;

(12) At4g34910_1089R: 5’- CTGGCTATTATCATCCGTTGCAATCA-3’ and At4g34910_815R: 5’- GGGACGGCCTCTTCCTTGTCATTAT-3’ for At4g34910;

(13) At4g36810_501R: 5’- ACGGCGGAGATCGTCGTTATCCATA-3’ and At4g36810_408R: 5’- GGTTGATTCTTCACCTCCGACGAGTT-3’ for At4g36810, and

(14) At3g57880_1974R: 5’- TAGCTCGTCGGGGTGGGCTGAGT-3’ and At3g57880_1911R: 5’- CGGTCTCCAACGGTAGTACCAGATA-3’ for At3g57880.

References

Llave, C., Xie, Z., Kasschau, K.D. and Carrington, J.C. (2002) Cleavage of Scarecrow-like mRNA targets directed by a class of Arabidopsis miRNA. *Science*, **297**, 2053-2056.

Xie, Z., Allen, E., Wilken, A. and Carrington, J.C. (2005) DICER-LIKE 4 functions in trans-acting small interfering RNA biogenesis and vegetative phase change in Arabidopsis thaliana. *Proc Natl Acad Sci U S A*, **102**, 12984-12989.

"CLASSIFY"

The input file is the sequencing result file in text (ASCII) format. The first column is the reads and the second column is the sequence. Below is a segment of an example input file:

```
98053 ACTTGTGGCCGAGGATGTTTCCGTCCTCGTATG
78904 TGTTTGGATTGAAGGGAGCTCTATCGTATGCCG
53996 CATTTGGATTGAAGGGAGCTCTATCGTATGCCG
```

Pseudocode for **CLASSIFY**:

```
   Input: Sequencing result file
   Result: Parse all sequences in the sequencing result file and output parsing results into output files
   Output: Output files and statistics on the screen
1  sum0 ← 0 (reads of useful sequences);
2  sum1 ← 0 ( reads of sequences without 5′ adapter match);
3  sum2 ← 0 ( reads of sequences without 3′ adapter match);
4  sum3 ← 0 ( reads of sequences with self-ligated sequences);
5  sum4 ← 0 ( reads of GFP);
6  Create and open output files used to store parsing result
7  while not at end of the input file do
8      Read in one new line from the input file. Denoted the sequence as seq and the read of the sequence as seq-reads
9      if The first 2 bases of seq is 'AC', 'CA', 'GT' or 'TG' then
10         seq ← seq without the first two bases.
11         if The last i bases of seq are the same as the first i bases of 3′ adapter then
12             seq ← seq without the last i bases
13         else
14             sum2 ← sum2 + seq-reads
15             Jump to step 8
16         end
17         if The last i bases of seq are the same as the first i bases of 3′ adapter then
18             sum3 ← sum3 + seq-reads (self-aliasing 3′ adapter has been found)
19             Jump to step 8
20         else if The last j bases of seq are the same as the first j bases of gfp18 OR gfp24 then
21             sum4 ← sum4 + seq-reads
22         else
23             Output seq to corresponding output file
24         end
25     else
26         sum1 ← sum1 + seq-reads
27         Jump to step 8
28     end
29 end
30 print sum0, sum1, sum2, sum3, sum4
31 Close output files
```

Source code for **CLASSIFY**:

```
#This software is a free software licensed under GNU General Public License version 3 or later.

# Cite this program as: Xiaopeng Qi, Forrest Sheng Bao and Zhixin Xie,
# Small RNA Deep Sequencing Reveals Role for Arabidopsis thaliana RNA-dependent
# RNA Polymerases in Viral siRNA Biogenesis, PLoS One, xxx: xxx, 2009

#usage: python classify.py <pool>
#no shell program embedding it

import string,os
from os.path import *
from numpy import *
from pylab import *

def classification(samples):
        #samples
        #[0]: segments
        #[1]: reads
        #[2]: class
        trim5, trim3, result = [],[],[]

        if samples[1][:2]=='AC':
                trim5=[samples[1][2:], int(samples[0]), 1]
        elif samples[1][:2]=='CA':
                trim5=[samples[1][2:], int(samples[0]), 2]
        elif samples[1][:2]=='GT':
                trim5=[samples[1][2:], int(samples[0]), 3]
        elif samples[1][:2]=='TG':
                trim5=[samples[1][2:], int(samples[0]), 4]
        else:
                return -1; # no 5' adaptor

        tail = "TCGTATGCCGTCTTCTGCTTG";

        windowL = 3; # initial slide window length
        windowU = len(tail); # slide window length to determine dumping a seq to trash3

        subject = trim5[0];
        should = 0 ;
        for j in xrange(windowL,windowU+1):#begin sliding from $windowL, don't exceed $windowU
                if subject[-j:] == tail[:j]:
                        trim3=[subject[:-j],trim5[1],trim5[2]];
                        should = 1;
                        if len(subject) > 34:
                                print subject,len(subject)
                        break;
                if should ==1:
                        print "if not break, let me know";
        if should == 0:
                return -2; #no 3' adapter

        match = 'TCGTATGCCGTCTTCTGCTTGTT';
        gfp18 = 'CATCCTATACGGCCACAA';
        gfp24 = 'TTGTGGCCGAGGATGTTTCCGTCC';

        if match.find(trim3[0])==-1 and gfp18.find(trim3[0])==-1 and gfp24.find(trim3[0])==-1:
                return trim3;
        elif match.find(trim3[0])>=0:
```

```
                return -3; # self-ligation
        elif gfp18.find(trim3[0])>=0 or gfp24.find(trim3[0])>=0:
                return -4; # GFP
        else:
                print match.find(trim3[i][0]);

        return 0; # no meaningful result

sum0,sum1,sum2,sum3,sum4 = 0,0,0,0,0

out = [];
for i in xrange(0,4):
        out.append([])
        for j in xrange(15,29):
                storefile = "./classified/s_6-A"+str(i)+"-"+str(j)+".txt"
                if (exists(storefile)):
                        os.remove(storefile)
                out[i].append(open(storefile,'a'))

f1=open(sys.argv[1],'r')
samples=[]
totalseq = 0;
while True:
        line = f1.readline()
        if len(line) == 0:
                break #EOF
        totalseq+=1;
        result = classification(line.split())
        if result==-1:
                sum1+=int(line.split()[0])
        elif result==-2:
                sum2+=int(line.split()[0])
        elif result==-3:
                sum3+=int(line.split()[0])
        elif result==-4:
                sum4+=int(line.split()[0])
        elif result==0:
                break;
        else:
                sum0+=int(line.split()[0])
                Ax=result[2]-1
                if len(result[0])<16:
                        box = 15-15
                else:
                        box = len(result[0])-15
                out[Ax][box].write(result[0]+'\t'+str(result[1])+'\t'+str(result[2])+'\n')

print "reads of useful sequences",sum0, double(sum0)*100/(sum0+sum1+sum2+sum3+sum4),"%";
print "reads of sequences without 5' adapter match:",sum1,\
 double(sum1)*100/(sum0+sum1+sum2+sum3+sum4),"%";
print "reads of sequences without 3' adapter match:",sum2,\
 double(sum2)*100/(sum0+sum1+sum2+sum3+sum4),"%";
print "reads of sequences self-aliasing 3' adapter:",sum3,\
 double(sum3)*100/(sum0+sum1+sum2+sum3+sum4),"%";
print "reads of GFP",sum4, double(sum4)*100/(sum0+sum1+sum2+sum3+sum4),"%";
print "reads of sequences:", sum0+sum1+sum2+sum3+sum4;

for i in xrange(0,3):
        for j in xrange(15,29):
                out[i][j-15].close()
```

"MAP"

The input file is the parsing result file in text (ASCII) format. The first column is the sequence. The second column is the reads. The last column is small RNA library Id. Below is a segment of an example input file:

```
TTTGGATTGAAGGGAGCTCTA 53996 2
TTTGGATTGAAGGGAGCTCTT 10708 2
TCGGACCAGGCTTCATTCCCC 7441 2
```

Pseudocode for **MAP**:

```
   Input: A parsing result file and a genome file in FASTA format
   Result: Find siRNAs in the genome with perfect match and return their locations
   Output: One output file recording searching results and two additional files for plotting
1  Create and open output files.
2  Load the genome sequence into the memory.
3  while not at end of the parsing result file do
4      Read in one new line from the parsing result file. Denoted the sequence as seq and the read of the sequence as
       seq-reads
5      if 18 <length(seq)< 25 then
6          find all occurence of seq on the genome
7          output all occurence to the file recording searching results
8          Repeat steps 6 and 7 to the complementary strand of the genome
9      Output data for visualizing those occurences
10 end
11 Close all files
```

source code for **MAP**:

```
#This software is a free software licensed under GNU General Public License version 3 or later.

# Cite this program as: Xiaopeng Qi, Forrest Sheng Bao and Zhixin Xie,
# Small RNA Deep Sequencing Reveals Role for Arabidopsis thaliana RNA-dependent
# RNA Polymerases in Viral siRNA Biogenesis, PLoS One, xxx: xxx, 2009

# run it in "classified" folder ;  python ../map.py s_6_tag-A3.txt ../tmvcg

import string,os
from os.path import *
from numpy import *
from pylab import *
import cPickle

def fastaread(filename):
#       print "Begin Read"
        seq0 = [];
        for line in filename.readlines():
                seq0.append(line.split());
        seq0 = seq0[1:];

        seq='';
        for i in xrange(0,len(seq0)):
                seq = seq + seq0[i][0];
        del seq0
#       print "Read done"
        return seq;

#f1=open("./results/"+sys.argv[1], 'r')
f1=open(sys.argv[1], 'r')
pool=[]
totalseq = 0;
for line in f1.readlines():
        pool.append(line.split());
f1.close()
#print pool
for i in xrange(0,len(pool)):
        pool[i][1] = int(pool[i][1])
        pool[i].append([])

genomename = sys.argv[2]
f2=open(genomename,'r')
genome = fastaread(f2)
f2.close()
genome = genome.upper();

#print pool
#print genome

#samples
#[0]: segments
#[1]: reads
#[2]: class
#[3]: position on mapped genome [location, S/AS]
#[4]: section # in 9 sections, from 0 to 8

# this function is changed in the new version
def addone(target, position, reads,direction):
```

```
        if target.get(position-1, 0) == 0:#padzero
                target[position-1] = 0
        if target.get(position+1, 0) == 0:#padzero
                target[position+1] = 0
#       target[position]=reads*direction
        target[position] = target.get(position, 0) + reads*direction
# end

def comp(genome):
        compi = {'A':'T', 'T': 'A', 'G':'C', 'C': 'G'}
        genomecom = list(genome)
        for i in xrange(0,len(genome)):
                genomecom[i] = compi.get(genome[i], '?')
        return ''.join(genomecom)
        """ find a gen in pool in genome via direction"""

def plotcount(genome,pool,direction):
        genomeplot = {}
        for i in xrange(0,len(pool)):
#               print "seq",i,"/",len(pool),"->",direction,sys.argv[1]
                segment = pool[i][0];
                if len(segment)>=18 and len(segment)<=25: # control the segment length
                        delimiter = 0;
                        while delimiter != -1:
                                if direction ==1:
                                        location = genome.find(segment, delimiter, len(genome));
                                        ####just once, we need to consider all conditions.
                                elif direction == -1:
                                        location = genome.find(segment[::-1], delimiter, len(genome));
                                        ####just once, we need to consider
                                if location >= 0:
#                                       addone(genomeplot, location, pool[i][1], direction)
# no need to zero pad if using stem plot
#                                       if genomeplot.get(location-1, 0) == 0:#padzero
#                                               genomeplot[location-1] = 0
#                                       if genomeplot.get(location+1, 0) == 0:#padzero
#                                               genomeplot[location+1] = 0
# end of zero pad

                                        genomeplot[location] = genomeplot.get(location, 0)\
                                         + pool[i][1]*direction
                                        pool[i][3].append([location,direction])
                                        delimiter = location + len(pool[i][0]);
                                elif location == -1: # can't find, then give up
                                        delimiter = -1
                                else:
                                        print "error"
        return genomeplot;

genomeplot = plotcount(genome,pool,1)
genomecomp = comp(genome)
genomecompplot = plotcount(genomecomp,pool,-1)

Aplotfilename = "../plotdata/"+sys.argv[2][3:]+sys.argv[1][:-4] + '.A.txt'
if (exists(Aplotfilename)):
        os.remove(Aplotfilename)
Aplotfile = open(Aplotfilename,'a')
cPickle.dump(genomeplot, Aplotfile)

ASplotfilename = "../plotdata/"+sys.argv[2][3:]+sys.argv[1][:-4] + '.AS.txt'
```

```
if (exists(ASplotfilename)):
        os.remove(ASplotfilename)
ASplotfile = open(ASplotfilename,'a')
cPickle.dump(genomecompplot, ASplotfile)

storefile = "../mapped/"+sys.argv[2][3:]+'.'+sys.argv[1]
if (exists(storefile)):
        os.remove(storefile)
out = open(storefile,'a')

#dump file written format
#[0]: segments
#[1]: reads
#[4,5]: position on mapped genome [location, S/AS] !!!written at last

OutputThreshold = 0 # output sequences of reads higher than OutputThreshold

for i in xrange(0,len(pool)):
        written = '';
        if pool[i][3]!=[] and pool[i][1]>OutputThreshold:
                written += pool[i][0] + "\t" + str(pool[i][1]) + "\t"
                for j in xrange(0,len(pool[i][3])):
        # write 1) segments, 2) reads 5) location on genome 6) sense or antisense $repeat 5 6$
                        written += str(pool[i][3][j][0]) + "\t" + str(pool[i][3][j][1]) + "\t"
                written += '\n'
                out.write(written)
```

"CUT"
The input file is a FASTA format file of TMV-Cg genome.
pseudocode for **CUT**:

Input: A FASTA file
Result: Generate 21-nt small RNAs from TMV genome
Output: Another FASTA file
Create and open output file.
Load the TMV genome sequence into the memory.
for $i = 1$ **to** *(length of TMV genome - 21)* **do**
    seq ← bases on the genome from the i-th base to the $(i + 20)$-th base
    Append *seq* and its FASTA header into output file
end
Close all files

source code for **CUT**:

```
#This software is a free software licensed under GNU General Public License version 3 or later.

# Cite this program as: Xiaopeng Qi, Forrest Sheng Bao and Zhixin Xie,
# Small RNA Deep Sequencing Reveals Role for Arabidopsis thaliana RNA-dependent
# RNA Polymerases in Viral siRNA Biogenesis, PLoS One, xxx: xxx, 2009

import sys,string
from numpy import *

def fastaread(filename):# this fastaread() is different from the one in miRNAclassify.py
        seq0 = '';
        for line in filename.readlines():
                if line[0]!= '>':
                        st = line.split()
                        st = st[0].upper()
                        seq0+=st;
        return seq0;

#read the first sequence
f1=open(sys.argv[1], 'r')
tmvpool = fastaread(f1)
#seq1=f1.readline()
#seq1=string.strip(seq1)

for i in xrange(0,len(tmvpool)-21):
        print '>',i,'| Random ';
        print tmvpool[i:i+21];
```
