# Supplementary figures and images for: Small RNA Deep Sequencing Reveals Role for Arabidopsis thaliana RNA-Dependent RNA Polymerases in Viral siRNA Biogenesis
Source: PLoS One. 2009 Mar 24;4(3):e4971. doi: 10.1371/journal.pone.0004971 (PMC2654919; doi:10.1371/journal.pone.0004971)

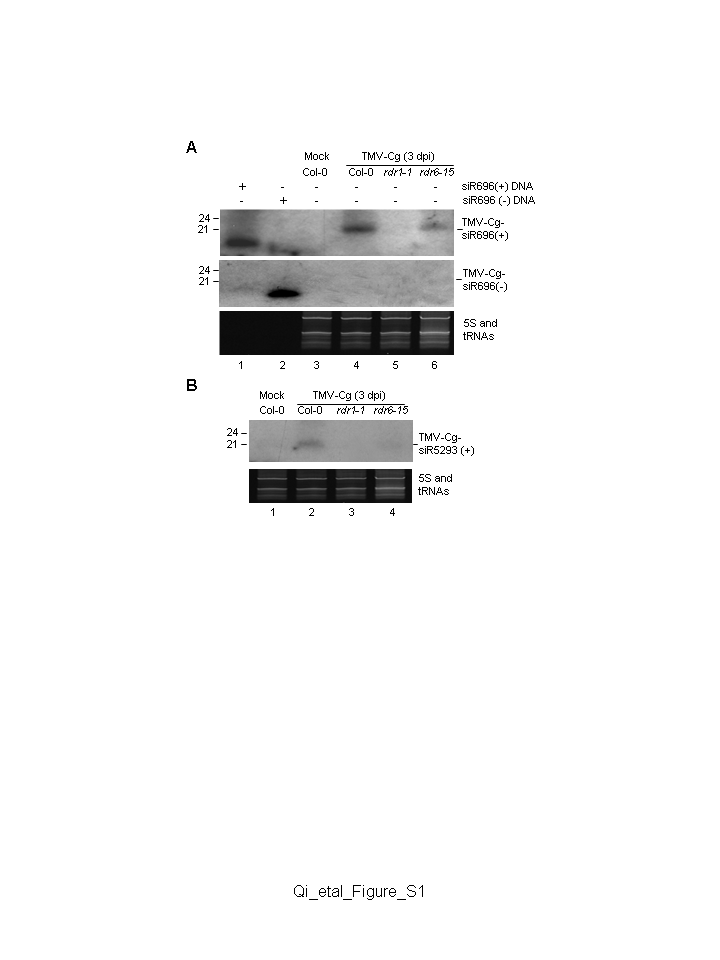

Supplement: Figure S1 — Detection of TMV-Cg-siRNAs by Northern blot assays. (A) Northern blot assays with probes specific to TMV-Cg-siR696 (+) (upper panel) and TMV-Cg-siR696 (−) (lower panel). DNA oligonucleotides with sequence corresponding to TMV-Cg-siR696 (+) and TMV-Cg-siR696 (−) were load as positive controls in lanes 1 and 2, respectively. (B) Northern blot assays with probes specific to TMV-Cg-siR5293(+). (0.12 MB TIF) [file pone.0004971.s003.tif]

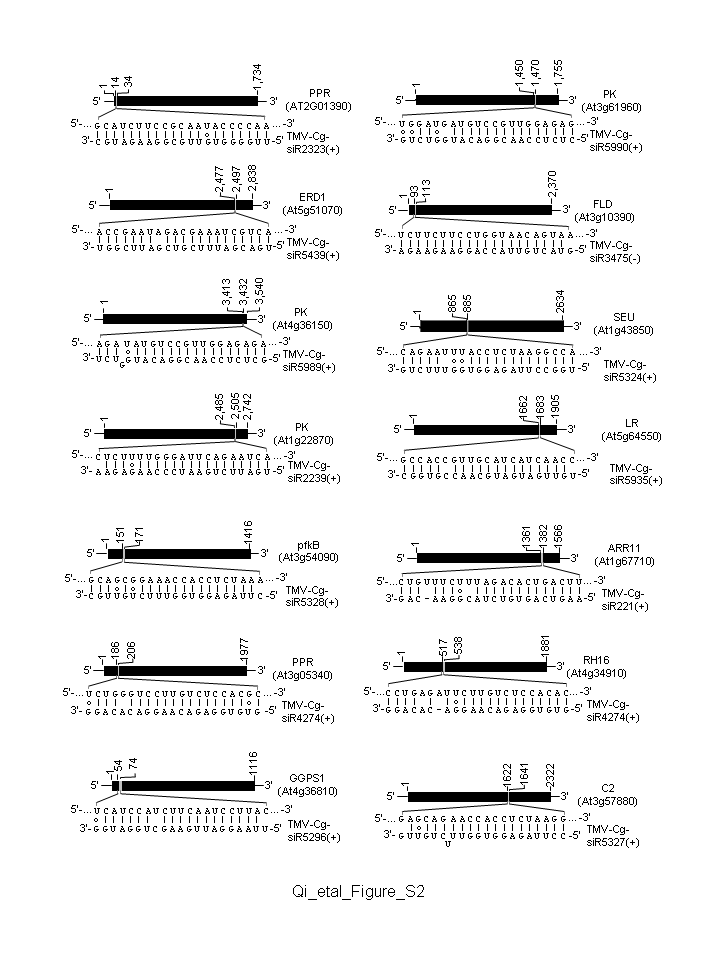

Supplement: Figure S2 — Additional predicted TMV-Cg-siRNA targets that were selected for validation by RLM-5′RACE. Sequence alignments between the TMV-Cg siRNAs and the respective predicted targets are shown. (0.09 MB TIF) [file pone.0004971.s004.tif]
